# Abstracts from Foreign Exchanges

**Published:** 1886-03

**Authors:** 


### ABSTRACTS FROM FOREIGN EXCHANGES.

DOLERIS and Poney (*compt. rend. hebd.*) come to the following conclusions:

I. Micro-organisms are sometimes found in the bladder, independent of albuminurea.

II. Albumen is found in about five per cent of pregnant women. In these strepto cocci are always found.

III. The blood of such women contains the same micro organisms.

IV. In some cases of eclampsia and albuminurea these cocci were present in blood and urine, in proportion to the gravity of the attack.

---

Godlee reports in *Med. Times and Gazette* two cases of incarcerated hernia in children, respectively five and six weeks of age. Both were operated on with good results.

---

In the Berlin Medical Association a woman was exhibited with a supernumerary mammary abscess in the axilla. Dr. Friedland recommended extirpation, as such erratic parts have the tendency to carcinomatous degeneration, which advice was supported by Prof. Bardeleben's statement.

